# Factors influencing delay in the diagnosis of colorectal cancer: a study protocol

**DOI:** 10.1186/1471-2407-7-86

**Published:** 2007-05-21

**Authors:** Magdalena Esteva, Maria Ramos, Elena Cabeza, Joan Llobera, Amador Ruiz, Salvador Pita, Josep M Segura, Jose M Cortés, Luis González-Lujan, the DECCIRE research group

**Affiliations:** 1 Primary Health Care Research Unit, Primary Health Care Mallorca District, Balearic Health Service, Reina Esclaramunda 9, 07003 Palma de Mallorca, Spain; 2 Deparment of Public Health, Balearic Department of Health, Font i Monteros s/n 07003 Palma de Mallorca, Spain; 3 Primary Care Centre Emili Darder, Primary Health Care Majorca District, Balearic Health Service, Avda Puerto Rico, 07007 Palma de Mallorca, Spain; 4 Epidemiology and Biostatistics Unit, Hospital Juan Canalejo, Galicia Health System, As Xubias de Arriba, 84 Hotel de Pacientes 7^a ^planta, 15006 La Coruña, Spain; 5 Primary Care Centre Lluis Saye, Catalàn Health Service, C/Torres i Amat, 8 08001 Barcelona, Spain; 6 Aragón Institute of Health Sciences. Avda Gómez Laguna 25 3^a ^planta, Zaragoza, Spain; 7 Serrería II Health Centre. Valencia Institute of Health, C/Pedro de Valencia 28, 46021 Valencia, Spain

## Abstract

**Background:**

Colorectal cancer (CRC) is the second most frequent tumor in developed countries. Since survival from CRC depends mostly on disease stage at the time of diagnosis, individuals with symptoms or signs suspicious of CRC should be examined without delay. Many factors, however, intervene between symptom onset and diagnosis. This study was designed to: 1) Describe the diagnostic process of CRC from the onset of first symptoms to diagnosis and treatment. 2) Establish the time interval from initial symptoms to diagnosis and treatment, globally and considering patient's and doctors' delay, with the latter due to family physician and/or hospital services. 3) Identify the factors related to defined types of delay. 4) Assess the concordance between information included in primary health care and hospital clinical records regarding onset of first symptoms.

**Methods/Design:**

Descriptive study, coordinated, with 5 participant groups of 5 different Spanish regions (Balearic Islands, Galicia, Catalunya, Aragón and Valencia Health Districts), with a total of 8 acute public hospitals and 140 primary care centers.

Incident cases of CRC during the study period, as identified from pathology services at the involved hospitals. A sample size of 896 subjects has been estimated, 150 subjects for each participant group.

Information will be collected through patient interviews and primary health care and hospital clinical records. Patient variables will include sociodemographic variables, family history of cancer, symptom perception, and confidence in the family physician; tumor variables will include tumor site, histological type, grade and stage; symptom variables will include date of onset, type and number of symptoms; health system variables will include number of patient contacts with family physician, type and content of the referral, hospital services attending the patient, diagnostic modalities and results; and delay intervals, including global delays and delays attributed to the patient, family physician and hospital.

**Discussion:**

To obtain a nonrestricted sample of patients with CRC we have minimized selection risk by identifying the patients from pathology services. A greater constraint may be associated with information sources based on clinical records. Due to inherent features of coordinated studies, it is important to standardize the collection of information.

## Background

Colorectal cancer (CRC) is one of the most common cancers in Western countries. In Spain, CRC incidence and mortality rates are close to the median values for European countries [1]. CRC survival rates in Spain are above the European average for both men and women and are greater for colon than for rectal cancer [2].

Survival of CRC patients depends mostly on their disease stage at the time of diagnosis. Most patients will be alive after 5 years if the tumor has not reached the intestinal wall (stage I). This rate decreases to 60% if the tumor has invaded regional lymph nodes (stage III) and to only 5–15% if the neoplasm has metastasized (stage IV) [2]. Thus, individuals with symptoms or signs suspicious of CRC should be examined without delay; however, many are not. Many aspects of the delay of diagnosis or treatment are poorly understood. Delay in diagnosis or treatment may be predictors of the stage and survival of CRC, but these results are controversial [3-8]. The majority of studies on CRC are not recent, dealt with small and restricted sample sizes and were limited to hospital settings.

Most studies use the term delay to describe the time elapsed between onset of the first symptom to diagnosis or treatment. Conceptually, diagnostic delay in cancer is a complex process involving patient behavior, physician attitudes, response of the health system, biology of the tumor cells, and interaction between host and tumor. Studies on delay tend to distinguish between patient and health system delays:

### 1. Patient delay

There are a number of reasons why a patient, facing a sign or symptom suspicious of malignancy, may decide not to visit a doctor. The patient may not be aware of the importance of symptoms [9], may be embarrassed to consult about them [9], may not relate them with the disease [10], or may fear a possible cancer diagnosis [11]. A family history of cancer together with a negative attitude to the medical profession can also be a reason for delay [12], as is a previous history of anxiety or depressive illness [13] Additional factors may include patient age, civil and social status, mistrust in doctors or lack of time to visit a physician [14,15]. Among symptom types, anemia is most frequently associated with longer delay [16,17], whereas multiple symptoms are associated with shorter delay [13]. In addition, a constitutional syndrome has been associated with a diagnosis made in the hospital emergency department, whereas low abdominal symptoms are more common in patients who undergo elective surgery [18].

### 2. Family doctor delay

The general practitioner plays an active role in cancer diagnosis, participating in nearly 63% [19]. His or her style of practice is important in recognizing CRC. A study on family doctors' clinical management of patients with gastrointestinal symptoms found that one third of patients did not have a physical examination, fewer than 50% underwent a digital rectal examination, and at least 90% of hospital referrals failed to include primary care findings [20]. Nonspecific symptoms, the absence of routine rectal examination, and patient reluctance to undergo rectal exploration may increase delay [21,22].

### 3. Hospital delay

Factors described as possible contributors to delay include specialist referrals' waiting lists, poor coordination, and complementary examinations. About 65% of patients affected by gastrointestinal tract cancers were initially diagnosed by a hospital emergency department, and over 50% had visited their general practitioners while having symptoms related to CRC [18].

It is clear that more information is needed on the effects of delay on CRC. The factors associated with diagnostic delay are poorly understood, regardless of whether they are related to the patient, to the family doctor or to the hospital setting. In Spain, several Regional Health Plans have highlighted problems in continuity of care and have advocated a reduction in time interval between suspicion of cancer and diagnosis or treatment. To achieve these objectives it is essential to know the characteristics of the CRC diagnostic process, to identify the delays that may occur at various stages, and to gain a better understanding of the factors associated with each type of delay.

## Objectives

1. To describe the diagnostic process of CRC from the onset of first symptoms until diagnosis and initial treatment in terms of initial symptoms and other symptoms appearing prior to and during the diagnostic process.

Family doctor participation in diagnosis: Proportion of cases diagnosed by the family doctor; visits per patient, physical examinations; complementary tests requested according to the nature of symptoms; type of referral to specialist and clinical information included in the referral.

Hospital specialist diagnostic procedures: hospital department, type of hospital; complementary examinations and their results (true positives and false negatives).

2. To establish the time intervals between first symptoms and diagnosis and/or treatment:

Patient delay: Time interval from the onset of first symptoms of CRC to first contact with a doctor.

Health system delay, separated into three time periods: from first contact with a doctor to referral to a specialist or emergency department; from specialist visit to diagnosis; and from diagnosis to treatment.

3. To identify factors associated with these delays:

Patient: sociodemographic variables; patient referred symptoms; attitude towards symptoms; confidence in the doctor; and family history of cancer.

Health system: primary care, including physical examination and clinical assessment by the family physician; and specialist care, including access to diagnostic tests, referral paths, type of hospital, type of department, number and type of complementary examination tests and their results.

Secondary objectives:

4. To evaluate the reliability and thoroughness of information about symptoms from patient interviews and primary care and hospital records.

5. To describe the variability in delay between different geographic areas and different hospitals included in the study.

## Methods

It is a descriptive and multicenter study in 5 participating health districts in Spain (Balearic Islands, Galicia, Catalunya, Aragón and Valencia), including 8 acute public hospitals and 140 primary health centers. Information will be collected from medical records and structured interviews with patients.

Subjects will be incident cases with histologically verified CRC (CIE9 153 and 154) diagnosed during 2006–07 in the study hospitals; patients have to be registered with a family doctor in the health center included in the study.

Exclusion criteria: Prevalent and/or recurrent cases; patients with multiple tumors; and patients diagnosed at private hospitals.

Cases will be identified by hospital pathologists. The doctor responsible for that patient will be contacted and informed of possible patient inclusion. Inclusion and exclusion criteria will be verified in the hospital clinical record, and written informed consent will be requested from the patient. Interviews will be carried out mainly during a patient's hospital stay, or at home if that is not possible. The sample size necessary for the whole multicentric project should be a minimum of 896 individuals to achieve the study objectives with an accepted 5% alpha error and an accuracy of one-tenth of the observed relative frequency of different variables. Moreover, the selected sample size will allow us to estimate symptom duration, despite the usual dispersion of a 'time' variable (usually a standard deviation over the mean). In addition, this sample size will permit to us to achieve objective number 3, to assess if delay is related to any of the described factors, with an alpha error ≤ 0.05 and a power ≥ 80%. Having at least 150 patients in each region will allow a precise estimation of geographical variations.

The number of persons included must be increased to 1,000, due to the inevitable collection of secondary and often incomplete information from primary care (PHC) and hospital (HC) clinical records.

A preinclusion patient form will be designed to hold patient pathology results together with exclusion criteria. A data collection book (DCB) will be prepared to include information about interviews, primary health care and hospital clinical records. A pilot study with 15 patients in each sub-project will be undertaken to standardize data collection and solve any conflicts. We will also carry out a training workshop for field workers and interviewers. Patient interviews will be centered mainly on CRC symptoms, perceived attitudes to initial symptoms and demographic data. Each patient will be asked how long he/she has been feeling unwell and the type of symptoms noted [23]. Symptoms spontaneously mentioned by the patient will be considered the initial symptoms for that patient and the date recorded. The patient will be asked to indicate whether he/she had experienced any additional symptoms summarized in a check-list. Symptoms perceived up to 2 years prior to the first consultation will be considered. Nonsymptomatic patients will be recorded as a casual finding. Information on examination test dates, results and clinical departments involved will be collected by reviewing medical records. Variables are summarized in Table 1. The study has received written approval from the Ethics Committee of Clinical Research of each participating region.

A database will be built with a unique numerical case code for each patient and checked for errors. Identifiable patient information will be kept dissociated. Each participating group will periodically send the patient data collection book to the coordinator node for centralized data entry. Number and reason for exclusion and missing cases will be noted.

Objective 1. A descriptive analysis of the key variables of the CRC diagnosis process will be presented as frequency distribution and 95% CI, and as mean and median.

Objective 2. It will be detailed total time delay; patient and health system delay and their contribution to total delay.

Objective 3. The relationship between different types of delay and the observed variables will be evaluated. The Chi-Square test will be used for qualitative variables, and Student's *t *test, ANOVA or non-parametric tests for quantitative variables. To assess the effect of the predictive variables on delay times, a survival analysis will be performed as proposed by Latour [24]. In contrast with follow up studies measuring survival, no data will be censored. Survival curves will be calculated by the Kaplan Meier method, and the log-rank test will be used to compare curves. To assess the independent effect of variables on delay times, a proportional risk analysis will be done using Cox regression. At the same time, the adequacy of performing a multiple level analysis, to evaluate the effects due to hospital center and District Health Authority, will be evaluated. Analysis will also be carried out separately for patients with cancers of the colon and rectum.

Objective 4. The thoroughness of symptom information given by patients will be compared with information obtained from the PHC and HC clinical records, and a frequency distribution of variables in each of the different sources of information will be presented.

Objective 5. The variability of different delay intervals will be evaluated by comparing median times and the inter-quartile range for the considered variables. Moreover, for each variable the proportional time variance of the study areas will be estimated, including hospitals with longer and shorter delay times and a graphic analysis.

## Discussion

We have minimized the selection risk by identifying study patients from pathology reports instead of at admission for surgery. In Spain the percentage of histological confirmation of CRC is greater than 96%. Nevertheless it is still possible that some patients would not be included if they had no biopsy results, due either to very advanced age or severe ill-health. A potentially more important constraint could be associated with information sources based on medical records. Use of secondary information could increase the risk of missing data for some variables. Nevertheless, we consider that the patient interview process will strengthen the comprehensiveness of information obtained. Due to the inherent features of coordinated studies it is very important to make a special effort to standardize data collection, since multiple observers in the different participating regions can introduce problems of inconsistency.

## Competing interests

The author(s) declare that they have no competing interests.

## Authors' contributions

ME, MR, EC, JLL, and AR participated in the design of the study. SP, JMS, JMC, and LG reviewed the study protocol and made suggestions that improved the design. All of these individuals are involved in the management of the study. ME, MR, EC, and JLL drafted the manuscript. All authors read, revised and approved the final manuscript.

## Pre-publication history

The pre-publication history for this paper can be accessed here:



## Figures and Tables

**Table 1:** Study measurements

Patient
Age, gender, civil status, education, family history of cancer, symptom perception, confidence in the family doctor, and number of consultations until the first contact and reason for it.
Tumour
- Site, hystological grade, TNM at diagnosis.

Clinical:
- First symptom, data of the first symptom and other symptoms.

Delay intervals:
Patient-delay:
- Time elapsed from the date the patient perceived the first symptom until the date of the first contact with a doctor as a result of the first symptom/s.
Health system-delay. This delay distinguishes the following phases:
- Time elapsed between the first contact with the health system (consultation with the family doctor, specialist, or emergency department) and until the date of referral to a specialist.-
- Time elapsed between referral to a specialist or emergency department and until the diagnosis. In this context diagnosis is understood as the date of the biopsy or direct surgery if that was the case.
- Time elapsed between diagnosis and treatment. In this context we consider surgical treatment. Failing on that chemotherapy or palliative care treatment as the first option.
Total delay: Sum of all previous delays. There will be a distinction between diagnostic and therapy delay.

**Health system**.
- Health professional who attended the patient at the first visit: Family doctor or specialist including which speciality.
- Teaching versus non-teaching primary care centre; urban versus rural ones and receiving hospital. – Hospital service that attended the patient in the various contacts during the diagnostic process.
- Number of consultations to the family doctor; to primary care emergency services; and to the specialist since the first contact. Consultations cancelled with family doctor and specialist.
- Complementary examinations done at the first visit: rectal exam; abdominal palpation; ultrasound; blood tests; X Ray films. Delay duration for each of the complementary tests. – Request and date of complementary tests at each contact prior to diagnosis. Results of complementary tests in order to exclude false negatives.
- Type of referral done by the family doctor to the specialist: preferential; programmed; emergency department. Diagnostic assessment and referral details in terms of presenting clinical picture, physical examinations.
